# Model-Based Equivalent Dose Optimization to Develop New Donepezil Patch Formulation

**DOI:** 10.3390/pharmaceutics14020244

**Published:** 2022-01-20

**Authors:** Woojin Jung, Heeyoon Jung, Ngoc-Anh Thi Vu, Gwan-Young Kim, Gyoung-Won Kim, Jung-woo Chae, Taeheon Kim, Hwi-yeol Yun

**Affiliations:** 1 College of Pharmacy, Chungnam National University, Daejeon 34134, Korea; tnzo12@o.cnu.ac.kr (W.J.); hy93.jung@o.cnu.ac.kr (H.J.); ngocanhzunie@o.cnu.ac.kr (N.-A.T.V.); 2 Life Science Research Institute, Daewoong Pharmaceuticals, Yongin-si 17028, Korea; pharmrich@daewoong.co.kr (G.-Y.K.); kchemist@daewoong.co.kr (G.-W.K.)

**Keywords:** donepezil, transdermal patch, equivalent dose optimization, model-based approaches

## Abstract

Donepezil patch was developed to replace the original oral formulation. To accurately describe the pharmacokinetics of donepezil and investigate compatible doses between two formulations, a population pharmacokinetic model for oral and transdermal patches was built based on a clinical study. Plasma donepezil levels were analyzed via liquid chromatography/tandem mass spectrometry. Non-compartmental analyses were performed to derive the initial parameters for compartmental analyses. Compartmental analysis (CA) was performed with NLME software NONMEM assisted by Perl-speaks-NONMEM, and R. Model evaluation was proceeded via visual predictive checks (VPC), goodness-of-fit (GOF) plotting, and bootstrap method. The bioequivalence test was based on a 2 × 2 crossover design, and parameters of AUC and C_max_ were considered. We found that a two-compartment model featuring two transit compartments accurately describes the pharmacokinetics of nine subjects administered in oral, as well as of the patch-dosed subjects. Through evaluation, the model was proven to be sufficiently accurate and suitable for further bioequivalence tests. Based on the bioequivalence test, 114 mg/101.3 cm^2^–146 mg/129.8 cm^2^ of donepezil patch per week was equivalent to 10 mg PO donepezil per day. In conclusion, the pharmacokinetic model was successfully developed, and acceptable parameters were estimated. However, the size calculated by an equivalent dose of donepezil patch could be rather large. Further optimization in formulation needs to be performed to find appropriate usability in clinical situations.

## 1. Introduction

Donepezil is frequently prescribed to treat Alzheimer’s disease (AD). The drug enhances cognitive function by inhibiting acetylcholine esterase (which degrades acetylcholine), thus increasing acetylcholine concentrations in the central nervous system. This is thought to prevent further degeneration of brain function. Currently, donepezil is approved for the symptomatic treatment of AD, characterized by a long half-life in physiological conditions [1,2]. In several clinical trials, cholinesterase inhibitors (including donepezil) slowed long-term AD development and exhibited suitable tolerability and safety profiles [3,4,5,6,7,8]. Donepezil 5–10 mg daily is an approved treatment for mild to moderate AD, and a dose of 10–23 mg daily can be used to treat moderate to severe AD.

If a drug is to be taken orally, patient compliance is a major issue. AD patients suffer from cognitive dysfunction. Thus, efforts have been made to increase the efficacy of donepezil by modifying the formulations and using extended-release tablets and transdermal patches. In a previous study on medication-nonadherent AD patients, those with transdermal donepezil patches tended to be more compliant than patients on tablets or capsules [9]. A transdermal patch prolonged treatment duration and patient adherence and stabilized drug levels between dosing intervals. Moreover, the medication was readily controlled by attaching or removing patches. In addition, avoidance of gastrointestinal incompatibility and the first-pass effect of the liver offers huge benefits [10].

The most common method to prove bioequivalence between two different formulations is to perform two-one-sided tests (TOST) followed by non-compartmental analysis (NCA) [11]. Pharmacokinetic parameters such as AUC (area under plasma concentration curve) and C_max_ (peak concentration) are calculated for both sides. Their ratios are considered for determination. If the PK variability of a drug is high, the risk of a type I error increases. Models such as the non-linear mixed effect model (NLME) can quantify and distinguish between different kinds of variabilities represented as between-subject variability (BSV) and within-subject variability (WSV). The model-based bioequivalent approach is widely accepted in cases such as clinical trials with sparse sampling points, uneven samplings between individuals caused by missing values, drugs with a long half-life and high variabilities, and steady state-inducing studies [12].

When developing an extended-release donepezil formulation, it is essential to derive C_max_ and AUC values in bioequivalent doses. In general, transdermal patches exhibit PK profiles that differ from those of orally administered drugs. In transdermal dosing, the drug absorption process into the systemic circulation is slower than oral dosing in general, resulting in smaller gaps between concentration peaks and troughs in a similar administration condition. It is thus difficult to harmonize the C_max_ and AUC of a transdermal patch, which reduces the bioequivalence margin. PK profiling of transdermal drug delivery is compromised by high-level variability. Individual skin characteristics and metabolic differences affect drug diffusion.

As mentioned previously, NLME model-based, equivalent dose optimization not only yields optimal doses for bioequivalent trials but also facilitates dosing in clinical trials; this is model-informed drug discovery. Here we compared test transdermal formulation and reference oral formulation that differed in terms of the dosing schedule. Bioequivalence data were derived from the secondary PK parameters of an iterative, simulated clinical study. Then the optimal extended-release donepezil formulation was further investigated.

## 2. Materials and Methods

### 2.1. Clinical Study Design

A randomized, open-label, two-treatment, two-sequence, two-period (period I and II, washout period in between), two-way crossover comparative bioequivalence study was conducted in healthy male volunteers. The PK models of the donepezil patch and oral formulations were derived from the clinical data sets of the TL/WZ/19/001141 study, which adhered to the guidelines of the Declaration of Helsinki, good clinical practice, and the International Conference on Harmonization. Twelve healthy subjects aged 18–45 years with a body mass index 18.5–30.0 kg/m^2^ were enrolled; all provided written informed consent. The clinical study protocol was approved by the institutional review board of Raptim Research Ltd. (Mumbai, India; IORG no. IORG0009526, DCGI reg. no. ECR/224/Indt/MH/2015/RR-18). Each subject was healthy on physical examination, medical history taken, and standard clinical laboratory tests. Exclusion criteria included any significant history or current evidence of malignancy; chronic infection; cardiovascular, renal, hepatic, ophthalmic, pulmonary, neurological, metabolic (endocrine), hematological, gastrointestinal, immunological, or psychiatric disease; and/or organ dysfunction. In addition, any history of allergy or hypersensitivity to or intolerance of donepezil or its excipients that, in the opinion of a clinical investigator, would compromise safety-triggered exclusion.

Donepezil dose for humans was decided from in vivo pre-clinical experiments with rats and minipigs in reference to in vitro skin permeability tests. The human equivalent dose for donepezil in the formulation was converted on the basis of body surface area.

We placed donepezil patches (108 mg/96 cm^2^) on the torsos or backs of six test subjects for 1 week, followed by a washout period of at least 21 days (to exclude any carryover effect). The controls received donepezil tablets (Aricept; 10 mg) once a day for 1 week, a total of seven times of dose.

### 2.2. Preparation of Donepezil Patch

The donepezil-loaded patch was prepared using the solvent casting method reported in the previous experiment, with a slight modification (Jung et al., 2019). Oppanol^®^ N100 (15%, *w*/*w*) was dissolved in toluene, while Oppanol^®^ B15 and B12 were prepared at a concentration of 50% (*w*/*w*) in the mixture of toluene and n-heptane (1:1, *w*/*w*). Kristalex™ F85 hydrocarbon resin was dissolved in toluene to obtain a final concentration of 80% (*w*/*w*). These solutions were mixed with homomixer (HIVIS MIX model 2P-03, PROMIX, Japan) at 50 rpm for 2 h by varying the ratio of each component of the donepezil-containing patch. Then, the final solution was left for 1 h to remove air bubbles and set to a thickness of 100 or 200 µm applied to the release liner (Silicone-coated polyester film 7300A, Loparex, Cary, NC, USA), and dried at 90 °C for 10 min using labcoater (CH-8156, Mathis AG, Oberhasli, Switzerland). Backing membrane (ScotchpakTM 1012 PET film, 3M, St.Paul, MN, USA) was attached to the dried patch, and the patch was cut into 10 cm^2^ (3.16 × 3.16 cm) or 20 cm^2^ (4.47 × 4.47 cm) sizes and packed in an aluminum foil pouch (ALLS 819202, Amcor, Gent, Belgium). All patches were sealed with a bag sealer (Lovero, Wenzhou, Zhejiang, China) and stored at room temperature before use.

In summary, Oppanol^®^ N100, B12, and B15 were used as an adhesive and Kristalex™ F85 hydrocarbon resin as a tackifier. BHT, LP300 or NMP, Kristalex™ F85, and mineral oil was used as stabilizer, permeation enhancer, tackifier, and plasticizer, respectively.

### 2.3. Quantitative Analysis of Donepezil in Plasma Using LC-MS/MS

Blood samples for donepezil assay were collected before dosing (within 2 h prior to administration) and 4, 8, 12, 24, 48, 70, 72, 74, 76, 80, 96, 120, 144, 168, 216, 264, and 312 h after administration. Samples were placed in prelabeled vacutainers with K3EDTA, centrifuged at 4000 rpm for 10 min at 5 °C, and the plasma stored at −80 °C. We determined plasma concentrations of donepezil using a high-performance liquid chromatography MS/MS system equipped with a pump (LC-30AD, Shimadzu, Kyoto, Japan) and an API3500 mass spectrometer. Donepezil and donepezil-d7 (internal standard) were separated on a reverse-phase C18 Gemini column (4.6 × 50 mm, 3 µm). The mobile phase was acetonitrile:5 mM ammonium acetate (90:10, *v*/*v*), and the flow rate was 0.6 mL/min. The oven temperature was 50 °C, and the injection volume was 5 µL. An electrospray ionization interface operating in the positive ion multiple reaction monitoring mode served as the ion source. The m/z values of the precursor/product ions of donepezil ranged from 380.2 to 91.0; the dwell time and collision energy were 200 ms and 48 V, respectively. The figures for donepezil-d7 were 387.2 to 98.1, 200 ms, and 45 V, respectively. The retention times of donepezil and IS were 1.32 and 1.28 min, respectively. The calibration curves were linear over the range 0.40–85.14 ng/mL. The curve precision and accuracy were 92.6–106.9 and 4.3%, respectively. The results of bioanalytical method validation were summarized in Supplementary Data (Tables S1 and S2 and Figure S1).

### 2.4. Model Development

Non-compartmental parameters were calculated with the R package ncappc [13]. We derived the C_max_, T_max_, AUC_last_, AUC_inf_, λ_z_, half-life (based on Λ-z), V_z_ (volume of distribution, observed), and CL (clearance, observed). The parameter distributions were evaluated, and the results were used to set the initial parameters for CA.

When performing CA, an adequate model structure had chosen to describe the drug concentration profile for each formulation. Parameter estimation was performed with the first-order conditional estimation with interaction (FOCE-I) method. Interindividual variabilities were modeled exponentially, additively, and proportionally. In deciding the error model for residual variability, additive, proportional, and combined error models were tested [14]. We evaluated the adequacy of the parameters by calculating the decrease in the objective function value. Data analyses were performed with NLME software NONMEM (version 7.4; Icon Development Solutions, Ellicott City, MD, USA) assisted by Perl-speaks NONMEM (PsN; version 5.2.6), R (version 4.1.1), and Rstudio (version 1.4.1717).

Model evaluation was performed with PsN, and the R packages xpose and xpose4. We drew goodness-of-fit (GOF) plots (including conditional weighted residuals) and used a visual predictive check (VPC) to compare model predictions against observations. In terms of nonparametric diagnostics, bootstrap (1000 replicates) was performed to evaluate the precision of the final estimates.

### 2.5. Simulation to Optimize Equivalent Dose

The bioequivalence test of the oral and patch donepezil was based on a 2 × 2 crossover design. We simulated data for 200 patients (100 each in the oral and patch groups). The integrated donepezil PK model was used for simulation. The principal parameters used to evaluate bioequivalence are the AUC and C_max_ [15]. The plasma concentration-time values from simulation were analyzed with R version 1.4.1 to obtain the AUC and C_max_ for each patient 672 to 840 h after administration (when the level of donepezil would be in the steady state). We used an iterative process using various patch doses to determine the dose that was bioequivalent to 10 mg oral donepezil. AUC and C_max_ ratios within 0.8–1.25 of the 90% confidence intervals (CIs) served as the bioequivalence criteria [15].

## 3. Results

### 3.1. Subject Demographics and NCA

Twelve healthy volunteers were enrolled, but only nine completed the study. Three subjects (two in the test group and one in the control group) withdrew (for personal reasons) in periods I and II. Data from the nine who completed both periods were used to develop the PK model and for statistical analyses (Table 1). The test and reference products were safe and well tolerated by fasting subjects. Four adverse events were reported during the study, and one was reported during the post-study safety assessment. There was no serious adverse event or major concern.

NCA was performed with data to 24 h after oral administration and with oral and patch data over the entire period. The C_max_, T_max_, and AUC values are listed in Table 2.

### 3.2. Model Development

We developed a two-compartment PK model to describe the elimination of orally administered donepezil. The FOCE-I method best described the drug concentrations. The drug amount put in the gut is transferred to the central compartment at a first-order rate (Equation (1)).

(1) $$\frac{dGUT}{dt}=-KA\cdot GUT,$$

where *GUT* stands for the drug amount disposed in the gut and *KA* for the rate of absorption in the gastrointestinal tract.

The drug disposed in skin by transdermal patch form passes through additional transit compartments; absorption by central compartments is thus delayed (Equation (2)). The drug amount that enters the skin (from the patch depot) was related to the patch dissolution percentage over time. We fit the relevant equation using in vitro data (Table S3). The amount of drug disposed on the skin is decided by coefficient driven from the difference between in vitro experiment and mean released amount measured by remains in the patch after clinical trial (Equation (3)).

(2) $$\begin{array}{c} \frac{dSKIN}{dt}=-KT\cdot SKIN,\\ \frac{dTR1}{dt}=KT\cdot SKIN-KT\cdot TR1,\\ \frac{dTR2}{dt}=KT\cdot TR1-KT\cdot TR2, \end{array}$$

(3) $$\begin{array}{c} Drug\;dissolution=\frac{78.257\cdot Duration}{Duration+8.481}\%\cdot Patch\;dose,\\ Disposed\;amount\;in\;skin=0.74\cdot Drug\;dissolution, \end{array}$$

where *SKIN* stands for the drug amount disposed in the skin from the formulation, *KT* for the rate of drug transfer/absorption to the central compartment. *TR*1 and *TR*2 represent the amount of the drug in the middle of transition. In Equation (3), Duration means the time with patch attached in hours.

**Figure 1:**
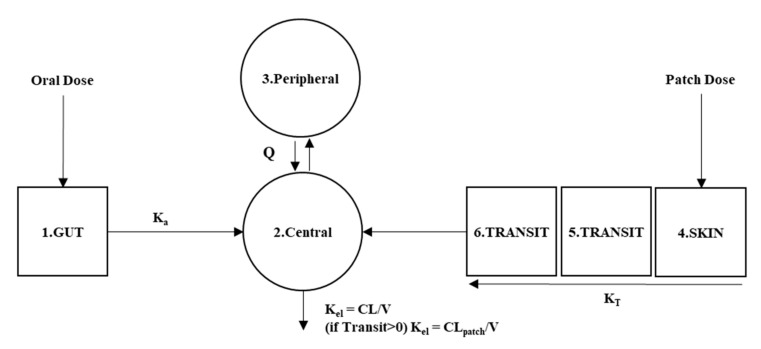
Compartmental scheme for oral and transdermal patch combined donepezil model used for bioequivalence test.

The central compartment receives drug amounts from both gut and skin, exchanges given amounts with the peripheral compartment, and eliminates at a rate of first-order kinetics (Equation (4))

(4) $$\begin{array}{c} \frac{dCENT}{dt}=KA\cdot SKIN+KT\cdot TR2+KPC\cdot PERI-KCP\cdot CENT-KE\cdot CENT,\\ KE=CL/V_{cent},\\ KCP=Q/V_{cent},\;KPC=Q/V_{peri}, \end{array}$$

where *CENT* and *PERI* stand for drug amount in central and peripheral compartment, *KE* for elimination constant, *CL* for clearance of oral and patch, *V_cent_* and *V_peri_* for central and peripheral compartments’ volume of distribution. *Q* stands for intercompartmental clearance between central and peripheral compartments. The patch and oral doses are eliminated in the same compartment, but the clearances differ when the drug remains in the patch (Figure 1).

**Figure 2:**
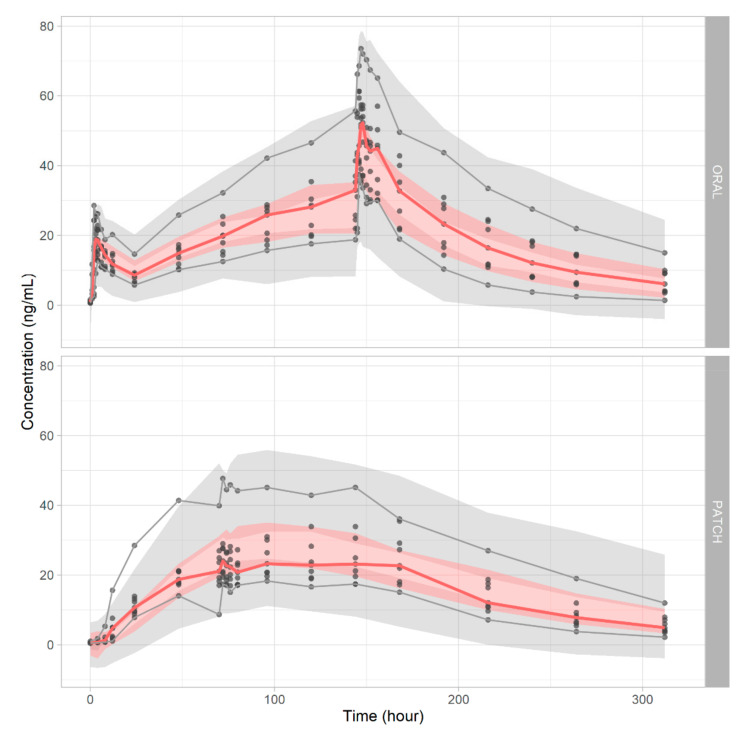
Visual predictive checks of donepezil in oral formulation (**upper panel**) and transdermal patch (**lower panel**).

The GOF plots of observations versus predictions showed that the model predictions were reasonable. Most conditional weighted residual values were included within ±2, and the trends lay around zero (Figure S2). The VPC revealed that the model simultaneously handled both oral and patch administration of the drug (Figure 2). With 1000 newly generated data set with the bootstrap method, 992 successful runs were observed, indicating the model’s robustness is sufficient. The model estimations are summarized in Table 3.

### 3.3. Simulation to Optimize Equivalent Dose

**Figure 3:**
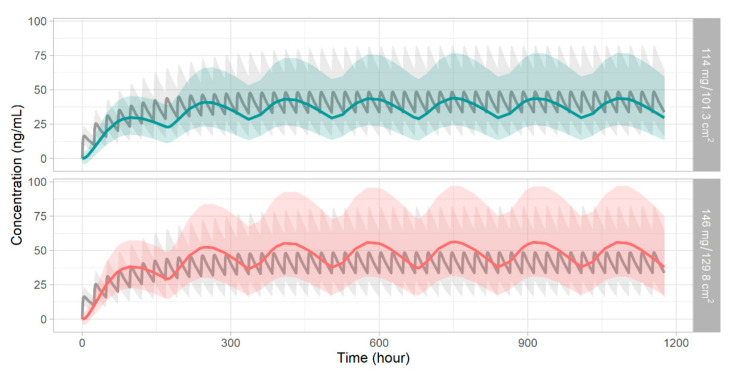
Simulated oral dose of donepezil 10 mg daily (gray line/area) and transdermal patch doses of 114 mg weekly (**upper**, green line/area) and 146 mg weekly (**lower**, red line/area). Lines: median predictions. Shaded areas: percentiles 5 to 95.

The bioequivalence results summarized in Table 4 indicate that weekly patch doses from 114 mg/101.3 cm^2^ to 146 mg/129.8 cm^2^ were equivalent to the administration of 10 mg donepezil orally. Typical values used to assess bioequivalence (AUC and C_max_ ratios) lay within 0.8- to 1.25-fold of the 90% CIs. The lower and upper 90% CI bounds for the AUC ratio were 85.61–97.06% for a 114 mg patch and 108.62–123.09% for a 146 mg patch. The C_max_ ratios were 82.07–91.51% and 102.98–114.88% for 114 mg and 146 mg donepezil, respectively. The simulations for each bioequivalent dose are plotted in Figure 3.

## 4. Discussion

The donepezil patch could replace the original oral formulation; the dosing frequency is thus reduced. The observed drug exposures (revealed by the C_max_ and AUC ratios) were slightly less than the predicted in vitro results, which indicates that adjustment of the patch dose may be required.

In this study, a population pharmacokinetic model of donepezil was developed as a two-compartment model for both oral and patch administration. Transit compartments were successfully applied for patch formulation to delay the arrival time to reach the central compartment. The same central compartment was used for both patch and oral dose, and its clearance was estimated as 10 L/h. A recent study using a population PK model of oral donepezil administration estimated an oral clearance of 12 L/h [16]. Another report stated that donepezil hydrochloride clearance was 9.65 L/h after administration of a 10 mg tablet [17]. The NCA PK parameters were 10 L/h (CLss) and 560 L (Vss) after oral administration of 10 mg drug. The Ka (absorption rate constant), Vc (central volume of distribution), Vp (peripheral volume of distribution), and Q (intercompartmental clearance) are listed in Table 2.

The VPC showed that the predictions were usually in agreement with the observations. However, the CIs for each percentile was rather wide, perhaps because of the intrinsic variability inherent in most of the transdermal formulation and estimation difficulties caused by flip-flop pharmacokinetics. It is thought that applying covariates of the study subject’s skin condition would help minimize the variability of the model. However, bootstrapping (1000 replicates) showed that the model was reliable and robust. The GOF data suggested that the model was accurate in terms of both population and individual predictions. Plots of the GOF of the conditional and individual weighted residuals (CWRES and iWRES) by time showed that the residuals were evenly dispersed around the predictions. The model was appropriate for further simulation study.

In a previous study, Yoon et al. 2020, described oral and patch formulation in two different population models. The administered drugs in oral and transdermal route were cleared in different spaces with different clearances. The study focused on separately developing a descriptive model for both oral and patch formulation [18].

In this research, different PK profiles from formulations were described with one integrated model and showed better agreement with the reported drug parameters. The model can handle complicated dosing plans such as giving an oral titration period in patch study. In vitro dissolution data is applied in deciding patch delivered dose so that the model can deal with further experiments of modifications on formulation. Overall, a more simplified and generalized model for interpreting oral and patch formulation was made.

Finally, we performed bioequivalence testing of oral (10 mg) and patch donepezil using a 2 × 2 crossover design (100 patients/group, 200 in total). The test was performed on the simulated secondary NCA parameters. Iteration revealed that the patch-equivalent drug dose lays between 114 and 146 mg (patch sizes of 101.3 and 129.8 cm^2^). Enhanced skin penetration or an increase in drug concentration would reduce the size of the patch, thus optimizing transdermal delivery of the drug by enhancing patient compliance.

For the first time, the inspection of appropriate patch doses satisfying the bioequivalence between two different formulations was performed. This model-informed bioequivalence assessment for different formulations was able to identify various kinds of variabilities and is expected to provide more accurate, interpretable data compared to the standard non-compartmental bioequivalence studies even in highly variable clinical situations.

## 5. Conclusions

To facilitate NCA-based bioequivalence testing, we built a population PK model for donepezil using data from nine healthy volunteers. We performed bioequivalence testing using secondary PK parameters derived from an iterative clinical simulation. A patch with 114–146 mg donepezil was equivalent to 10 mg oral donepezil.

## Figures and Tables

**Table 1:** Demographic and baseline data (n = 09) of evaluable subjects.

Parameter	Age (yrs)	Weight (kg)	Height (cm)	BMI (kg/m^2^)
Mean	29.56	66.11	165.94	24.00
SD	2.88	9.54	7.02	2.99
Median	30.00	63.10	168.40	24.67
Min	24.00	55.70	150.60	19.78
Max	33.00	80.90	173.60	27.33
%CV	9.73	14.43	4.23	12.46
Sex				
Male	09 (100%)			
Female	00			
Race				
Asian	09 (100%)			
Other	00			

Three subjects (two in the test group and another in the control group) withdrew from the study (for personal reasons) in periods I and II.

**Table 2:** NCA parameters by study group.

Parameters Mean (SD)	Oral (0–24 h)	Oral (0–312 h)	Patch (0–312 h)
C_max_	20.26 (5.24)	53.86 (12.60)	28.62 (8.70)
T_max_	3.11 (1.17)	146.33 (0.87)	106.67 (41.76)
AUC_last_	286.62 (75.47)	6111.08 (2245.90)	5285.59 (1892.27)
AUC_inf_	575.02 (242.57)	6873.40 (2772.54)	5909.34 (2291.77)
Λz	0.03 (0.01)	0.02 (0.01)	0.01 (0.0017)
HL	22.25 (4.95)	37.74 (11.60)	71.17 (11.95)
Vz	590 (130)	-	1080 (280)
Vss	-	560 (140)	-
Cl	20 (10)	10 (0.0030)	10 (0.0031)

SS: steady state, NS: non-steady state, C_max_: peak concentration, T_max_: peak time, AUC: area under curve, HL: half-life (based on Λ-z), Vz: volume of distribution (observed), Cl: clearance (observed), Vss: volume of distribution (steady state, observed).

**Table 3:** The final parameter estimates of the donepezil integrated PK model.

Parameter		Estimates (RSE%)	IIV (RSE%) [Shr%]	IIV in CV%
Oral	Ka (1/h)	0.0497 (25%)	0.00968 (28%) [51%]	9.9%
	CL (L/h)	10 (9%)	0.13 (12%) [0%]	37.3%
	Vc (L)	26.2 (35%)	0.198 (33%) [42%]	46.8%
	Q (L/h)	15.6 (33%)		
	Vp (L)	562 (11%)		
Patch	Kt (1/hr)	0.027 (9%)	0.02 (37%) [31%]	14.2%
Total	**Residual variability (RSE%)**			
	Additive error	2.89 (13%)		
	Proportional error	0.0795 (29%)		
	OFV	1443.703		

RSE: relative standard error, Shr: shrinkage, IIV: interindividual variability, CV: coefficient of variation, Ka: absorption rate constant, Vc: central volume of distribution, Vp: peripheral volume of distribution, Q: inter-compartmental clearance, CL: clearance on central volume, Kt: rate constant for transit compartment.

**Table 4:** Results of the bioequivalence study on the simulation of the two donepezil formulations.

Range	Parameter	AUC	Cmax
Minimum (114 mg/101.3 cm^2^)	CI 90% (Lower-Upper)	85.61–97.06	82.07–91.51
	RT Ratio (%Ref)	91.16	86.66
Maximum (146 mg/129.8 cm^2^)	CI 90% (Lower-Upper)	108.62–123.09	102.98–114.88
	RT Ratio (%Ref)	115.63	108.77

CI: confidence interval, RT Ratio: equivalence ratio of test formulation to reference formulation, “Test” refers to patch donepezil dose, “Reference” refers to oral donepezil dose.

## Data Availability

Pharmacokinetic profiles of both oral and transdermal patch is available in Supplementary Materials (Figures S3 and S4).

## Supplementary Materials

The following are available online at https://www.mdpi.com/article/10.3390/pharmaceutics14020244/s1, Table S1: Intra- and inter-day precision and accuracy values, Table S2: Stability of donepezil in human plasma, Table S3: Dissolution profile of oral and transdermal patch formulation, Figure S1: Representative chromatography of donepezil in human plasma ((A) double blank, (B) zero blank, (C) LLOQ, (D) sample obtained xx h after transdermal administration, and € sample obtained xx h after oral administration), Figure S2: Goodness-of-fit plot of model (A): Observation vs. population prediction, (B): Observation vs. individual prediction, (C): individual weighted residuals vs. individual predictions, (D): conditional weighted residuals vs. individual prediction, Figure S3: Pharmacokinetic profile of subjects with oral administration, Figure S4: Pharmacokinetic profile of subjects with transdermal patch administration.
