# Supplementary material for: Model-Based Equivalent Dose Optimization to Develop New Donepezil Patch Formulation
Source: Pharmaceutics. 2022 Jan 20;14(2):244. doi: 10.3390/pharmaceutics14020244 (PMC8880217; doi:10.3390/pharmaceutics14020244)
Supplement: Supplementary file 1 [file pharmaceutics-14-00244-s001.zip › pharmaceutics-1524890-supplementary.pdf]

pharmaceutics

MDPI

Supplementary Materials: Model-Based Equivalent Dose Optimization to Develop New Donepezil Patch Formulation

Woojin Jung, Heeyoon Jung, Ngoc-Anh Thi Vu, Gwan-Young Kim, Gyoung-Won Kim, Jung-woo Chae, Taeheon Kim and Hwi-yeol Yun

The donepezil was validated in human plasma using LC-MS/MS was performed in accordance with the U.S. Food and Drug Administration guidelines regarding bioanalytical method validation. The matrix effect, recovery and process efficiencies of donepezil and IS in human plasma were evaluated at three QC sample concentration levels. The matrix effect of donepezil was 98.91% to 111.7%, and the recovery was 89.10% to 89.16% for analyte and 94.30% to 95.77% for internal standard.

Table S1. Intra- and inter-day precision and accuracy values.

Spiked Concentration (ng/mL)	Measured Concentration (ng/mL)	Precision (CV (%))	Accuracy (RE (%))
Intra-batch (n = 50)			
0.40 (LLOQ)	0.42 ± 0.0700	16.67	5.00
1.20 (LQC)	1.19 ± 0.0447	3.76	−1.83
29.91 (MQC)	30.45 ± 0.2932	0.96	1.81
64.05 (HQC)	64.15 ± 0.6731	1.05	0.16
Inter-batch (n = 86)			
0.40 (LLOQ)	0.41 ± 0.0625	15.24	2.50
1.20 (LQC)	1.23 ± 0.0680	5.53	2.50
29.91 (MQC)	30.18 ± 0.4766	1.58	0.90
64.05 (HQC)	63.64 ± 0.9654	1.52	−0.64

Table S2. Stability of donepezil in human plasma.

Storage Conditions (n = 5)	Spiked Concentration (ng/mL)	% Stability in Human Plasma
27 h at RT	1.20	98.33 ± 1.46
	64.05	101.14 ± 0.71
102 h at auto-sampler	1.20	111.67 ± 0.77
	64.05	100.02 ± 0.85
1 month at -20°C	1.20	97.50 ± 2.12
	64.05	101.22 ± 0.79
Freeze-thaw 3 cycles	1.20	98.33 ± 1.46
	64.05	101.14 ± 0.71

Table S3. Dissolution profile of oral and transdermal patch formulation.

**Oral Formulation**	Sampling time points	0, 5, 10, 15, 30, 45, 60
	Dissolution %	0, 29.3, 73.1, 84.5, 92.6, 96.7, 98.9
**Patch Formulation**	Sampling time points	0, 2, 4, 6, 8, 10 ,12, 18, 24, 30

Dissolution %	0, 18.7, 24.2, 29.5, 36.1, 42.2, 49.7, 53.2, 57.8, 60.4

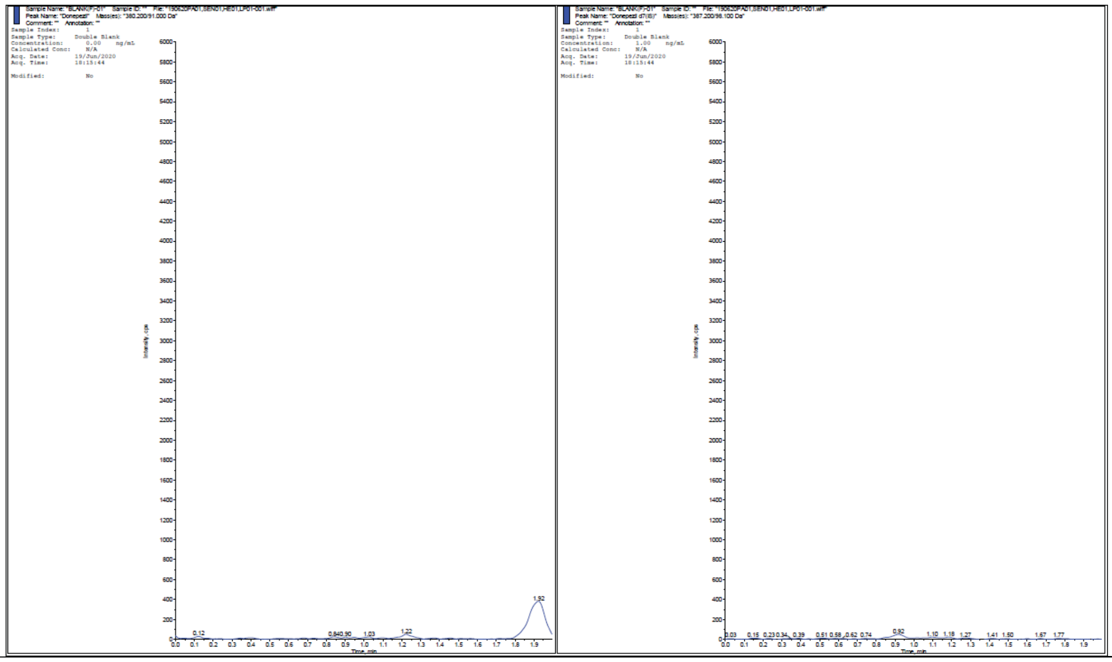

(A)

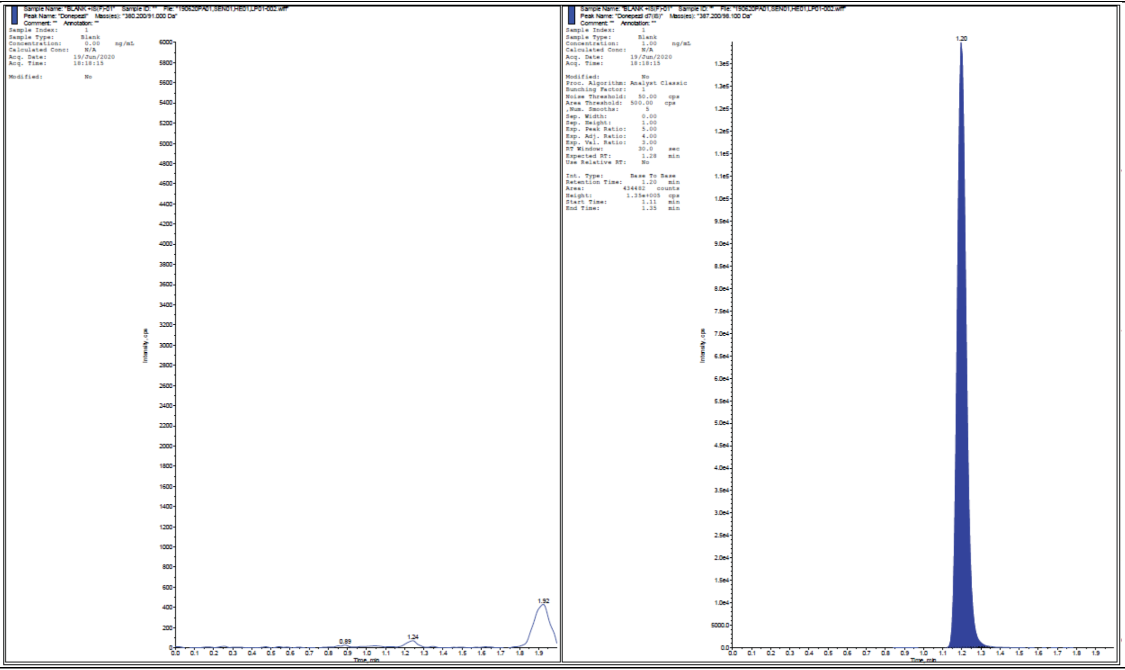

(B)

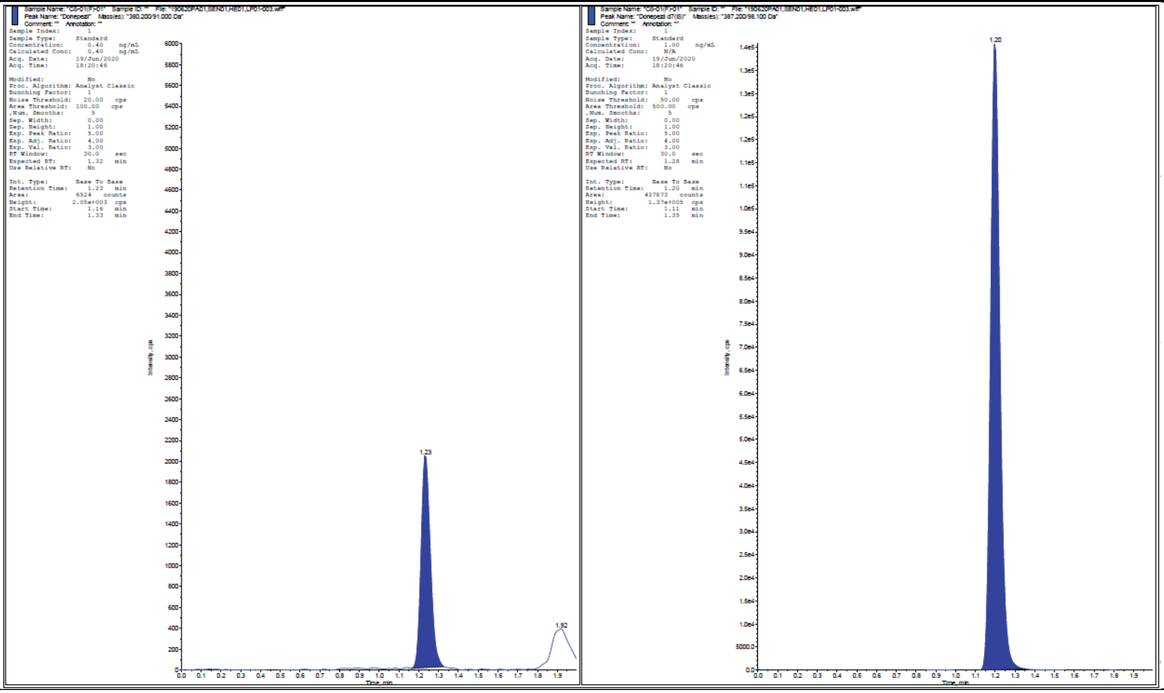

(C)

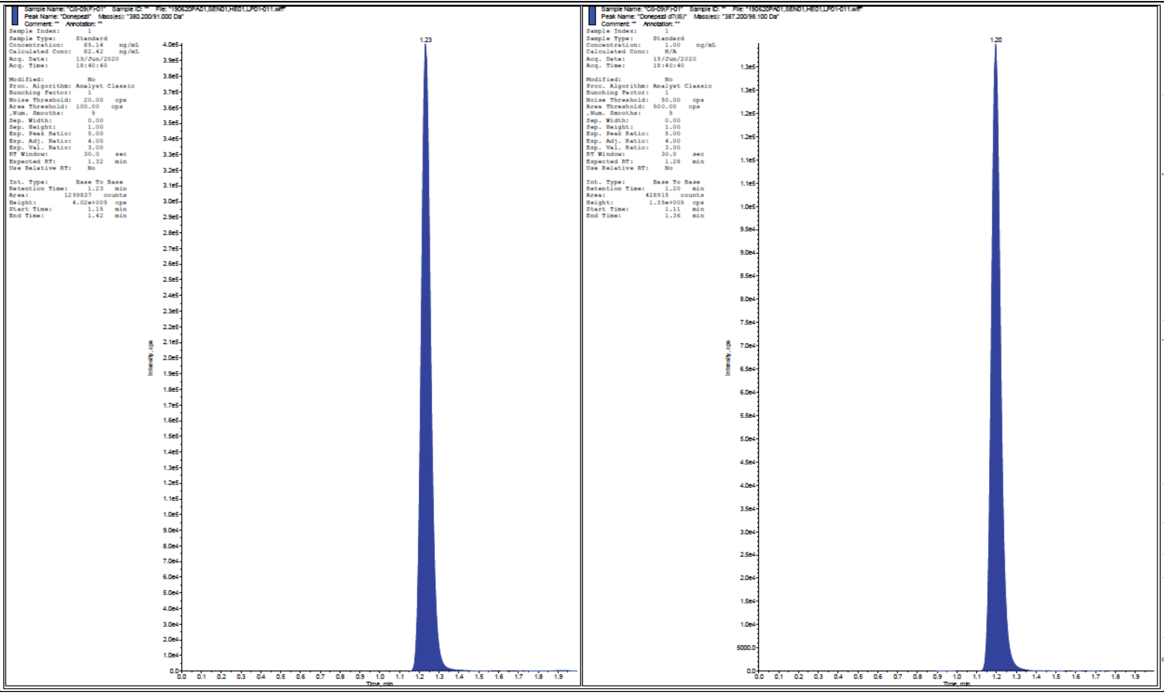

(D)

Figure S1. Representative chromatography of donepezil in human plasma ((**A**) double blank, (**B**) zero blank, (**C**) LLOQ, (**D**) sample obtained xx h after transdermal administration and € sample obtained xx h after oral administration).

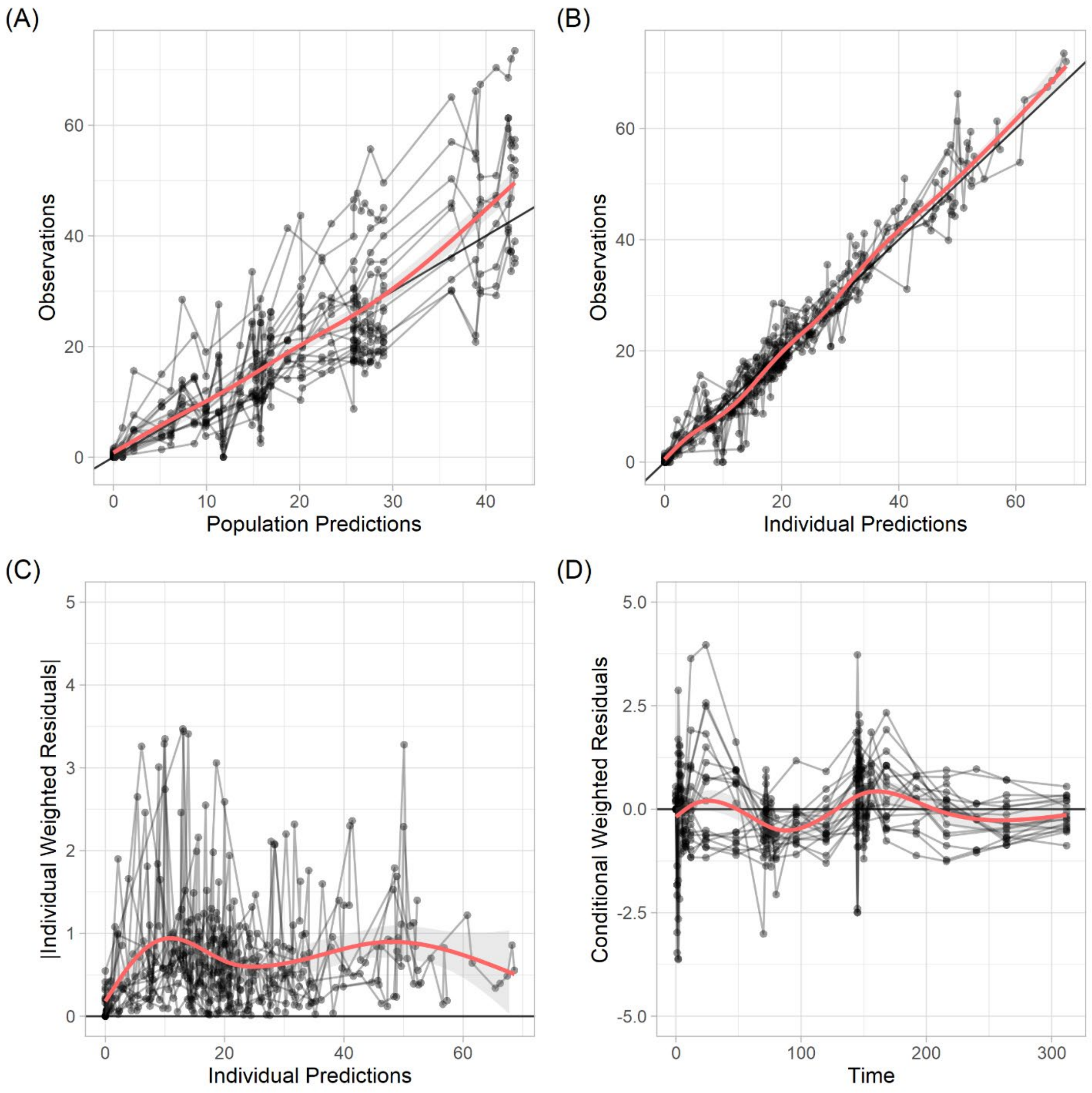

Figure S2. Goodness of fit plot of model (**A**): Observation vs population prediction, (**B**): Observation vs individual prediction, (**C**): individual weighted residuals vs individual predictions, (**D**): conditional weighted residuals vs individual prediction.

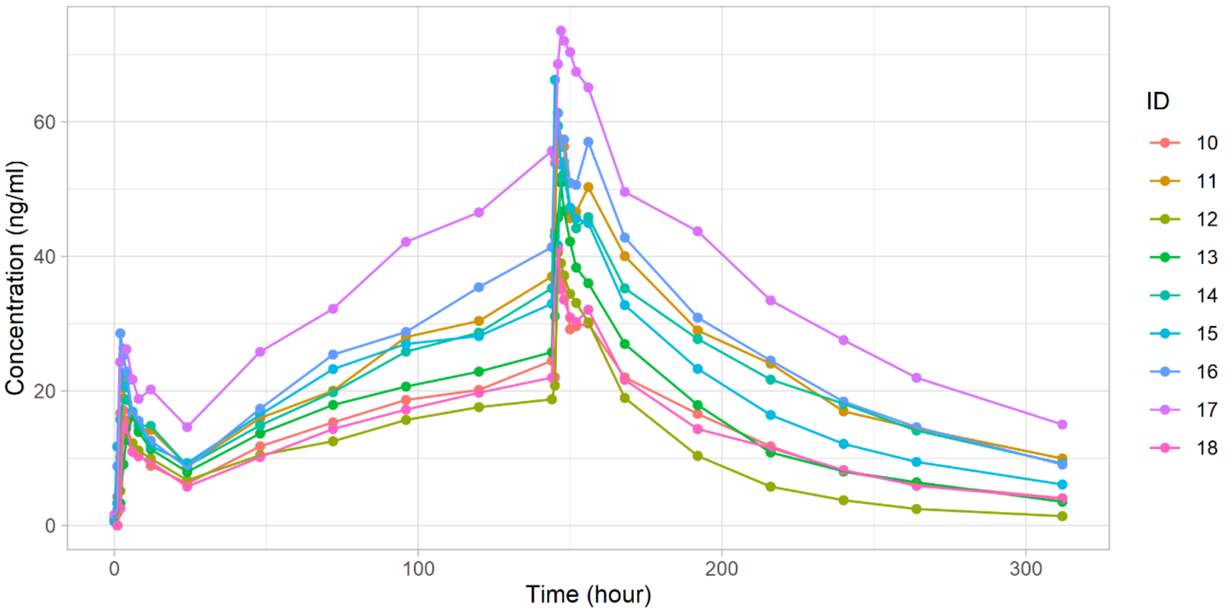

Figure S3. Pharmacokinetic profile of subjects with oral administration.

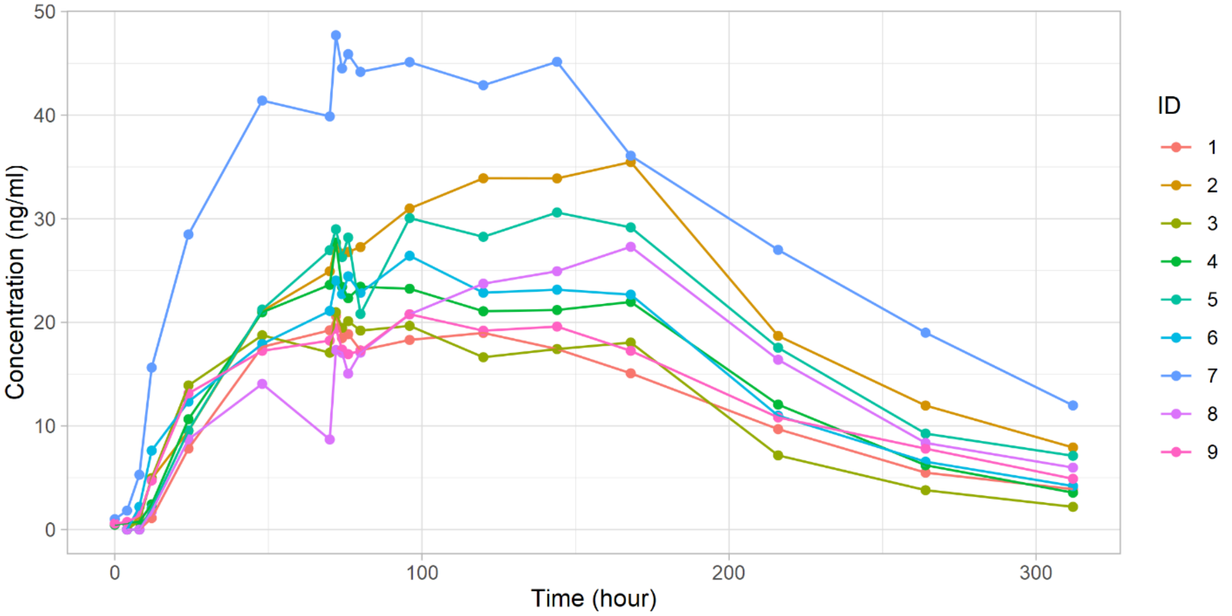

Figure S4. Pharmacokinetic profile of subjects with transdermal patch administration.
